# Characteristics of Opioid-Users Whose Death Was Related to Opioid-Toxicity: A Population-Based Study in Ontario, Canada

**DOI:** 10.1371/journal.pone.0060600

**Published:** 2013-04-05

**Authors:** Parvaz Madadi, Doris Hildebrandt, Albert E. Lauwers, Gideon Koren

**Affiliations:** 1 Division of Clinical Pharmacology and Toxicology, Department of Pediatrics, Hospital for Sick Children, Toronto, Ontario, Canada; 2 The Office of the Chief Coroner of Ontario, Toronto, Ontario, Canada; The James Cook University Hospital, United Kingdom

## Abstract

**Background:**

The impact of the prescription opioid public health crisis has been illustrated by the dramatic increase in opioid-related deaths in North America. We aimed to identify patterns and characteristics amongst opioid-users whose cause of death was related to opioid toxicity.

**Methods:**

This was a population-based study of Ontarians between the years 2006 and 2008. All drug-related deaths which occurred during this time frame were reviewed at the Office of the Chief Coroner of Ontario, and opioid-related deaths were identified. Medical, toxicology, pathology, and police reports were comprehensively reviewed. Narratives, semi-quantitative, and quantitative variables were extracted, tabulated, and analyzed.

**Results:**

Out of 2330 drug-related deaths in Ontario, 58% were attributed either in whole or in part, to opioids (n = 1359). Oxycodone was involved in approximately one-third of all opioid-related deaths. At least 7% of the entire cohort used opioids that were prescribed for friends and/or family, 19% inappropriately self-administered opioids (injection, inhalation, chewed patch), 3% were recently released from jail, and 5% had been switched from one opioid to another near the time of death. Accidental deaths were significantly associated with personal history of substance abuse, enrollment in methadone maintenance programs, cirrhosis, hepatitis, and cocaine use. Suicides were significantly associated with mental illness, previous suicide attempts, chronic pain, and a history of cancer.

**Significance/Conclusion:**

These results identify novel, susceptible groups of opioid-users whose cause of death was related to opioids in Ontario and provide the first evidence to assist in quantifying the contribution of opioid misuse and diversion amongst opioid-related mortality in Canada. Multifaceted prevention strategies need to be developed based on subpopulations of opioid users.

## Introduction

Non-medical use of prescription opioids has culminated in a public health crisis in many North American jurisdictions. The impact of this crisis has been powerfully illustrated by the dramatic increase in opioid-related deaths [1]–[6]: in 2008, prescription opioids were involved in 14,800 accidental deaths in the United States [4]. That there has been a parallel increase in the consumption of prescription opioids and deaths related to opioid drugs is not in dispute. Previous studies of Ontarians whose cause of death was related to opioids have deduced several relationships between opioid prescription practices and opioid-related deaths. Firstly, the introduction of long-acting oxycodone to the provincial formulary has been singled out as an important contributor to the increase in opioid-related morality in this province [2]. Secondly, regions/municipalities within the province with a high incidence of opioid-related deaths per capita have high opioid prescription utilization [7]. Thirdly, opioid-related deaths appear to be concentrated amongst patients treated by physicians who prescribed opioids more frequently [8], and high doses are significantly associated with an increase risk of mortality [9]. Such discoveries have subsequently shaped new provincial strategies to help curb and prevent this epidemic [10]–[12].

However, we need more individualized evidence and insight on how and why opioid-related deaths occur in order to develop holistic, inclusive, and multifaceted preventative strategies towards this issue. For example, indicators of opioid diversion and misuse amongst those whose cause of death was opioid-related in Canada has not been evaluated, despite data illustrating the enormity of these considerations in the United States [13], [14]. We aimed to identify patterns and characteristics amongst opioid-users whose cause of death was related to opioid toxicity in the province of Ontario between the years 2006 and 2008.

## Methods

This study was approved by the Office of the Chief Coroner of Ontario and the Research Ethics Board of the Hospital for Sick Children in Toronto, Canada. The Office of the Chief Coroner of Ontario conducts research in the public interest for the purpose of preventing future deaths and disseminates the findings of this research to the public on a regular basis. The authority to collect and analyze information about deaths in order to prevent future deaths in the public interest is provided in section 15(1) of the Ontario *Coroners Act*. The ability to conduct examinations and analysis appropriate in the circumstances is provided by section 28(2) of the Ontario *Coroners Act*. The Research Ethics Board of the Hospital for Sick Children granted a waiver of consent for next of kin in this research study based on the following three necessary conditions: 1) The objectives of the research cannot be reasonably accomplished without using personal health information, 2) There is a public interest in this research while protecting the privacy of individuals and 3) There are adequate safeguards to protect the privacy of individuals. It was also considered that given the subject group was deceased individuals, contacting the families would cause distress. The data collected in this study was coded and analyzed anonymously. No personal identifiers were collected.

Ontario is the most populous province in Canada with an estimated population of 12.69 million in 2006, rising to 12.93 million in 2008. Under the Ontario *Coroners Act*, all sudden and unexpected deaths, and/or deaths thought to be from any cause other than disease must be reported to the Coroner's Office from anywhere in the province of Ontario. The coroners' death investigations involve classification of the cause of death as well as the manner of death according to five categories: homicide, accident, suicide, natural, and undetermined. Particularly relevant to this study were the classification of accidental death (due to an occurrence, incident, or event that happens without foresight or expectation), suicide (an intentional act of omission or commission in a person knowing the probable consequence of what he/she is about to do), and undetermined [(a) there is no evidence for any specific classification; (b) there is equal evidence, or a significant contest, among two or more classifications, or (c) a death is a suicide that does not meet a high degree of probability].

The records of the Office of the Chief Coroner of Ontario were examined and all deaths coded as drug and alcohol-related between 2006 and 2008 were reviewed. From these files, all deaths in which opioids had been identified by the coroner were isolated. Medical, toxicological, pathological, and police reports compiled as part of the coroner's report were comprehensively reviewed. Narratives, semi-quantitative, and quantitative variables were extracted, tabulated, and analyzed. Indicators of opioid misuse and diversion were assessed amongst all opioid-related fatalities. A nonmedical route of drug administration was determined from coroner, police, and/or autopsy findings (i.e. death scene investigations, puncture sites on body, patch debris). Indicators of diversion were based solely on narratives found in coroner and police reports. These reports were informed by coroner and police analysis of prescription records, prescription bottles, interviews with family and friends, consultations with healthcare providers, and other circumstantial data gathered as part of the death investigation.

In addition, factors which have been validated for predicting risk of opioid misuse or addiction in patients were assessed in this cohort. In particular, data on gender, age, psychological disease, and personal history of substance abuse as reported in the Opioid Risk Tool (ORT) [15] were evaluated. Descriptive statistics (mean, standard deviation, median, minimum, and maximum) were calculated. Pearson's Chi-square, Fischer Exact, Student T-test, and Mann-Whitney U test were used as appropriate.

## Results

**Figure 1:**
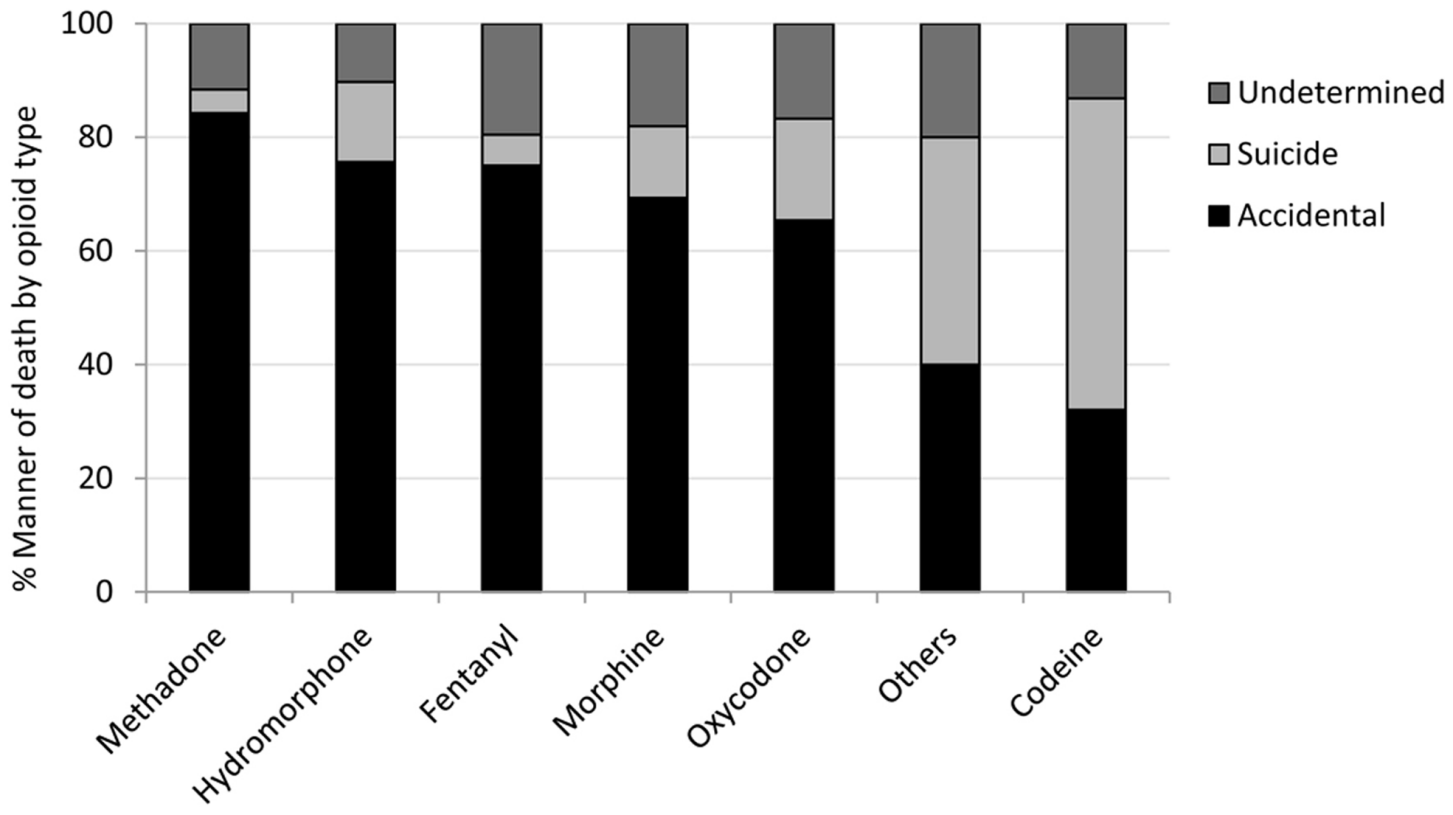
Manner of death per opioid type. This graph illustrates the relative proportion of accidental, suicide, or undetermined manners of death per opioid type. The graph represents all single opioid-related deaths in Ontario, Canada between the years 2006 and 2008 (n = 1040 decedents).

In Ontario, there were 2330 individuals whose cause of death was deemed to be drug-related between 2006 and 2008. Opioids were implicated in 58% (n = 1359) of these cases. Individuals whose cause of death was opioid-related were significantly younger, were disproportionately male, and their manner of death was significantly more likely to be deemed accidental **(** **Table 1** **)**. Conversely, when the deaths were due to drug toxicity but were non-opioid related, a significantly higher proportion of suicidal overdoses were observed **(** **Table 1** **)**. Oxycodone (35%) was involved in approximately one-third of all opioid-related deaths, followed by morphine (28%) and methadone (15%) **(** **Table 2** **)**. Oxycodone was also associated with the highest proportion of both accidental deaths and suicides. However, when analyzing the proportion of the types of deaths (accident, undetermined or suicide) within each opioid type, methadone had the highest relative percentage of deaths which were accidental (84%), while codeine had the lowest proportion of accidental deaths (32%) **(** **Figure 1** **)**.

**Table 1:** Comparison of demographic characteristics between opioid-related mortalities and non-opioid drug related mortalities in Ontario for the years 2006, 2007, and 2008.

Demographic Characteristics		Opioid deaths (n = 1359)	Non-opioid deaths (n = 971)	p-value
Gender	Male	867 (63.8%)	572 (58.9%)^a^	0.03
Age	Median (IQR)	44 (35–51)	46 (37–54)	^+^<0.001
	Range	[16–89]	[14–94]	
Coroner Death Classification				
Accident		924 (68.0%)	437 (45.0%)	<0.001
Undetermined		221 (16.3%)	163 (16.8%)	0.73
Suicide		214 (15.7%)	371 (38.2%)	<0.001

a Two values not available. All tests were performed by Pearson's Chi-square unless otherwise indicated. ^+^Mann-Whitney U-test. Note: 20 files were not available for assessment or contained missing information.

**Table 2:** Opioid-related deaths in Ontario between the years 2006 and 2008, by type of opioid.

A. Deaths in which a single opioid has been implicated (n = 1040)	
*Opioid*	*Number (% of 1040)*
Oxycodone	358 (34.4)
Morphine (major)	283 (27.2)
*6-MAM (heroin) confirmed*	*48 (17)*
*Codeine (minor)*	*47 (17)*
Methadone	146 (14)
Fentanyl	92 (8.8)
Hydromorphone	78 (7.5)
Codeine (only)	63 (6.1)
Other	20 (1.9)

B. Type of opioids detected across all opioid-related deaths (n = 1359)*	
*Opioid*	*Number (% of 1359)*
Oxycodone	480 (35.3)
Morphine	380 (28)
Methadone	210 (15.5)
Codeine	155 (11.4)
Fentanyl	140 (10.3)
Hydromorphone	129 (9.5)
Meperidine/normeperidine	31 (2.3)
Others	29 (2.1)

* One person could have used multiple opioids.

6-MAM: 6-monoacetyl morphine (heroin metabolite).

Opioid self-administration by inappropriate route of injection, inhalation, or patch ingestion was identified in 19% of all individuals (**Table 3**). Prior to death, 101 individuals (7.4%) had used opioid medications which were diverted and had belonged to their partners, family members or friends **(** **Table 3** **)**. Signals of double-doctoring, purchasing opioids from a street source, and of health workers diverting opioids for personal use were detected in 2.1%, 2%, and 0.6% of all cases, respectively **(** **Table 3** **)**.

**Table 3:** Indicators of diversion and opioid misuse amongst opioid-related deaths in Ontario (n = 1353).

Descriptor	Number (%)
Health worker diverting for personal use	8 (0.6)
Double-doctoring (intentional)	28 (2.1)
Opioid was known to be purchased from a street source	26 (2)
Using someone else's opioid(s) n = 101	
Live family member/partner	57 (4.2)
Deceased family member	5 (0.4)
Friend	39 (2.9)
Inappropriate route n = 263	
Intravenous use	219 (16)
Chewing fentanyl patch	14 (1.0)
Inhalation/Other	30 (2.2)

Prior to death, 50 individuals (3.7%) had been switched to a more potent opioid medication **(** **Table 4** **)**; an additional 9 individuals had received opioids for acute pain while in methadone programs and another 7 had their methadone dose adjusted prior to the fatality. Further analysis was performed to identify factors specifically associated with accidental versus suicidal overdose with opioids. Individuals whose manner of death was accidental were younger and were more likely to have a history of substance abuse as compared to those committing suicide **(** **Table 4** **)**. These individuals were significantly more likely to be enrolled in methadone programs and had a higher incidence of cirrhosis, hepatitis, and cocaine use prior to death **(** **Table 4** **)**. In 242 accidental deaths, the deceased had contact with a healthcare worker (physician or pharmacist) less than 5 days before death **(** **Table 4** **)**. Individuals who committed suicide were significantly more likely to have received a larger number of medications, to have had previous suicide attempts, depression and/or other psychiatric morbidities, and to have had a history of cancer and chronic pain **(** **Table 4** **)**.

**Table 4:** Health characteristics amongst Ontarians whose cause of death was related to opioids; compared by manner of death.

Health and disease characteristics	Accidents(n = 921)	Suicides (n = 215)	OR	95% CI	P-value
Cancer history	24 (2.6)	17 (7.9)	3.21	1.7–6.1	0.0002
Disability/wheelchair bound	43 (4.7)	14 (6.5)	1.42	0.8–2.7	0.265
Lung/airway disease	106 (11)	22 (10)	0.88	0.5–1.4	0.593
Diabetes history	60 (6.5)	17 (7.9)	1.23	0.7–2.2	0.464
Hepatitis	123 (13)	3 (1.4)			#<0.0001
HIV	(12) 1.30	0.00			#0.138
Cirrhosis	70 (7.6)	5 (2.3)			#0.003
+ADD, OCD, bipolar, and/or schizophrenia	83 (9)	36 (17)	2.1	1.4–3.2	0.0005
	^n = 918^	^n = 209^			
Depression	167 (18)	112 (53.6)	5.2	3.8–7.1	<0.0001
	^n = 918^	^n = 209^			
Previous suicide attempts	37 (4.0)	72 (33)	12.0	7.8–18.6	<0.0001
**Substance abuse-related features**					
Age (16–45)	563 (61)	74 (35)	2.9	2.1–3.9	<0.0001
Personal history of alcohol abuse	221 (24)	25 (12)	2.3	1.5–3.6	0.0001
	^n = 918^	^n = 209^			
Alcohol detected	302 (33)	64 (30)	0.86	0.6–1.2	0.392
Personal history of illegal drug abuse	486 (53)	17 (8.1)	8.3	5.3–12.8	<0.0001
	^n = 918^	^n = 209^			
Cocaine/benzoylecgonine detected	308 (34)	22 (11)	4.34	2.7–6.9	<0.0001
Personal history of prescription drug abuse	457 (50)	35 (16.7)	4.9	3.3–7.2	<0.0001
	^n = 918^	^n = 209^			
**Prescribing-related features**					
Last dispensed medication and/or health care visit (days)	*2 (1–5)	*5 (2–13)			++0.0003
	^n = 242^	^n = 65^			
Number of known prescribed medications	*2 (0–5)	*4 (2–7)			++<0.0001
	^n = 705^	^n = 164^			
*Opioid indication*: Methadone program	79 (8.6)	4 (1.9)			#0.0001
*Opioid indication*: Chronic Pain	307 (33)	111 (52)	2.14	1.6–2.9	<0.0001
*Opioid indication*: Acute pain	66 (7.2)	10 (4.7)	0.63	0.3–1.25	0.184
*Recent opioid switch (n = 50) in overall cohort*: Methadone 15 (1.1);	Oxycodone 10 (0.7);	Fentanyl 10 (0.7);	Hydromorphone 4 (0.3);	Morphine 3(0.2);	Others 8 (0.6).

All tests were performed by Chi-square and reported as number (percent) unless otherwise indicated.

+ Attention Deficit Disorder, Obsessive-Compulsive Disorder, or Bipolar, Schizophrenia with or without depression.

++ Mann-Whitney U-test.

# Fisher Exact test.

* These values are reported as median (inter-quartile range).

There were 46 individuals (∼3% of opioid-related deaths between 2006 and 2008) whose death was temporally related to custody or release from a correctional facility **(** **Table 5** **)**. Over 90% of these deaths were accidental, 20% occurred while the individual was in custody, and a further 43% occurred within 7 days of release from jail **(** **Table 5** **)**. Forty percent of these individuals were found to have injected opioids, and cocaine was detected in 65% of these cases **(** **Table 5** **)**.

**Table 5:** Opioid-related deaths in Ontario which were temporally associated with release from a correctional institution or under custody (n = 46).

Descriptor	Number (%)
Male	41 (89)
Age	*36 (28–43)
Timeframe of detainment (days)	* +90 (3–180)
Accidental death	43 (93.5)
Drugs administered by injection	18 (39)
Alcohol detected	8 (17)
Cocaine and/or benzoylecgonine detected	30 (65)
History of mental illness	7 (15)
**Main opioid detected**	
Morphine	12 (26)
Oxycodone	16 (35)
Methadone	11 (24)
Others	7 (15)
**Days released from jail**	
In custody	9 (19.6)
1–7 days	20 (43.5)
>1–4 weeks	9 (19.6)
“Recent” (not defined)	8 (17.4)

* These values are reported as median (inter-quartile range).

+ Based on seven cases in which information pertaining to the length of detainment was available.

## Discussion

Amongst all deaths in Ontario that were due to drug intoxication/overdose, a far greater proportion of accidental deaths were identified when opioids were involved. Conversely, in non-opioid related deaths, a significantly higher proportion of suicide was observed. This demonstrates the potential role that opioids play in the genesis of accidental deaths, where unforgiving margins of prescribing or ingesting errors can be lethal and necessitate great caution on the part of prescribers and users.

Oxycodone was involved in approximately one third of opioid-related deaths. This drug has been associated with the rising number of opioid deaths in the province of Ontario [2], [11]. In parallel however, there have also been dramatic increases in the use of fentanyl, hydromorphone, and methadone in Canada over the same time frame [16]–[18]. When we evaluated the manner of death based on opioid type, a high proportion of accidental deaths occurred amongst methadone, hydromorphone, and fentanyl users. This and other data [19]–[21] suggests that a range of prescription-opioids constitute this public health crisis, particularly as more individuals are enrolled in treatment programs for prescription-opioid addiction [22]–[24], or are switched from oxycodone to other potent opioids.

One in five individuals whose cause of death was related to opioids utilized an inappropriate route of drug administration such as injection, inhalation, or chewing pills or patches. Diversion occurred in 7.4% of the deaths, including 8 cases in which healthcare workers diverted opioids for their personal use. Opioids were known to be purchased from the street in approximately 2% of opioid-related deaths, however identifying the root source of diverted opioids beyond what can be gleaned from interviews, witnesses, and the immediate death scene investigations is limited in coroner-led investigations. Notwithstanding, these figures assist with quantifying the contribution of opioid abuse and illicit opioid diversion to mortality in Ontario, and buttresses arguments that support greater utilization of drug monitoring and other surveillance systems [25] directed at promoting appropriate use while discouraging abuse and diversion.

Switching to a more potent opioid, adding an opioid to someone taking methadone, or adjusting a methadone dosage, was associated with accidental opioid-related deaths. These are practices which present healthcare providers with unique and potentially lethal outcomes if not done with great caution. Enlisting the aid of collaborative expertise such as pharmacists, pharmacologists or addiction medicine consultants might assist practitioners faced with these situations. Recent data suggests that approximately 18% of individuals in the methadone program in Ontario received at least one prescription for non-methadone opioids [26].

The Opioid Risk Tool (ORT) was applied posthumously for a limited number of parameters including personal history of substance abuse, age, and mental illness. A personal history of substance abuse, particularly illicit or prescription drugs, and a younger age were more likely to be associated with accidental overdose. For suicidal deaths, depression and mental illness were strongly correlated with deliberate overdose. In addition, a history of previous suicide attempts was known in one-third of those who committed suicide with opioids (versus just 4% of those whose manner of death was accidental). Yet the ORT is specifically designed to assess the risk for opioid abuse or addiction. While there may be an overlap between predictors for opioid abuse or addiction and predictors for opioid-related death, the sheer magnitude of individuals who succumb to opioid-related toxicity necessitates the need to identify individuals at risk specifically for opioid overdose. Such an assessment should consider a specific question directed at whether the potential recipient of an opioid prescription has ever attempted suicide in the past.

Opioid-related deaths occurred while individuals were incarcerated and/or shortly after release. Almost all of these deaths were accidental, and 43% occurred within one week of release. In addition, 39% utilized injection as the preferred administration route, and 65% had evidence of cocaine or its metabolites present. A high rate of acute drug-related mortality amongst prison populations in the immediate post-release period has been described in other settings [27]–[29]. A contributing factor is decreased tolerance during incarceration. Upon release, individuals may utilize previous doses based on their beliefs regarding their own tolerance. Almost 90% of post-release substance abuse deaths in Australia, England and Wales, and Switzerland involved opioids [27]. Our findings point to the need for opioid substitution treatment interventions during incarceration and a coordinated effort between prison and public health systems to provide education to inmates on these issues.

Our current analysis is limited by several factors. All data in this study were obtained from coroners' reports, but there is variability among coroners in how death investigations are conducted across the province. Furthermore, toxicology testing in Ontario for coroners' cases is not standardized and depends on the individual scientists in charge of the case, the history of drug use as collected by the coroner, and the volume and type of samples available. The drug fentanyl, for example, was not part of the regular toxicology screen at the time of these fatalities and needed to be specifically requested [30]. Thus, certain drugs may be underrepresented in our study cohort based on these differences. Finally, we did not include an in-depth assessment of drug-interactions associated with this study cohort. Drug interactions and an examination of genetic mechanisms which may predispose certain individuals to these fatalities will be the subject of a subsequent investigation by our team.

It is evident that opioid-related mortality is associated not only with high risk prescribing, but also with personal characteristics of individuals who receive or use this class of drugs. Previous studies which illustrate the short-term safety and efficacy of prescription opioids excluded patients with substance abuse disorders [31]; but in this present population-based study, there was an overrepresentation of individuals with a history of drug abuse. In particular, one in five decedents had self-administered opioids inappropriately. We also identified other vulnerable Ontarians including those involved in the correctional system, those with previous history of suicide, those whose doctors had recently switched their opioid medication, and those involved in a methadone program. Our multifaceted findings point to the need for diverse prevention strategies to be developed based on subpopulations of opioid users.
